# Supplementary material for: Better sturdy or slender? Eurasian otter skull plasticity in response to feeding ecology
Source: PLoS One. 2022 Sep 29;17(9):e0274893. doi: 10.1371/journal.pone.0274893 (PMC9521905; doi:10.1371/journal.pone.0274893)
Supplement: S1 Fig — Points represent 9 sampling locations for which diet composition was available, colours indicate genetic clusters. The first two axes of a Principal Component Analysis for percentage of seven prey categories (marine fish, freshwater fish, crustaceans, amphibians, reptiles, birds, mammals, and insects) are used to summarise variation between points. (DOCX) [file pone.0274893.s002.docx]

**S1 Fig. Spatial variation in the UK otters’ diet.** Points represent 9 sampling locations for which diet composition was available, colours indicate genetic clusters. The first two axes of a Principal Component Analysis for percentage of seven prey categories (marine fish, freshwater fish, crustaceans, amphibians, reptiles, birds, mammals, and insects) are used to summarise variation between points.

**
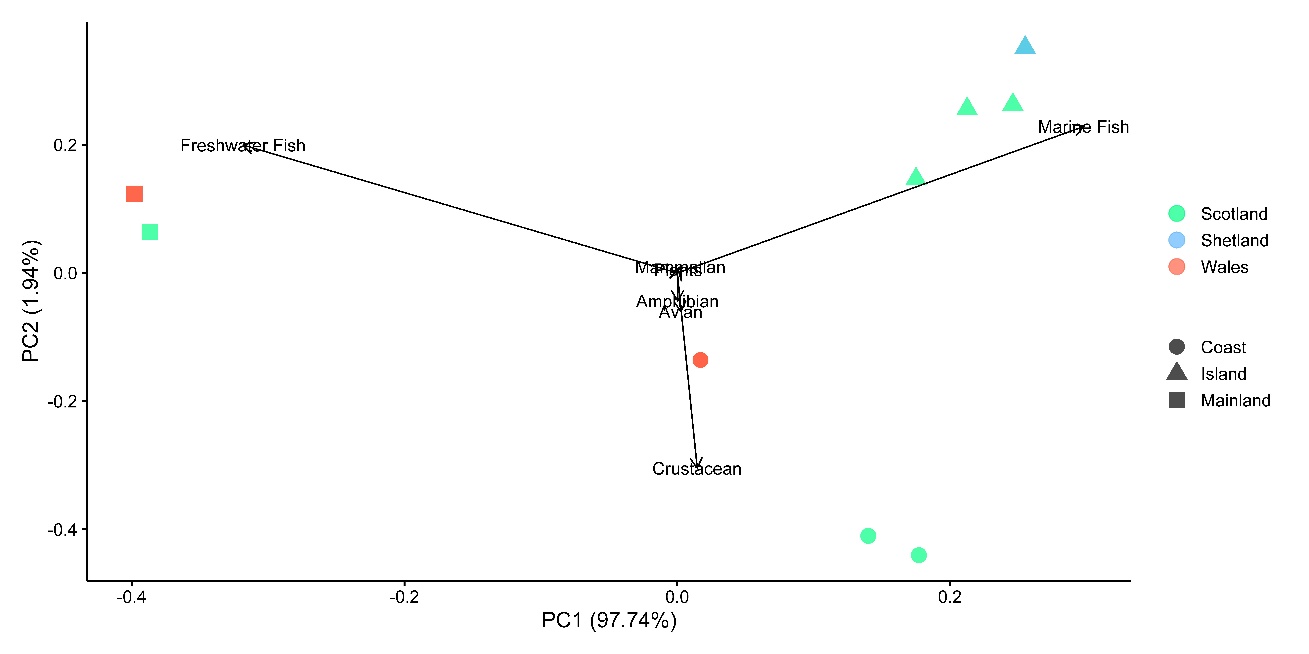
**
