# Supplementary material for: Better sturdy or slender? Eurasian otter skull plasticity in response to feeding ecology
Source: PLoS One. 2022 Sep 29;17(9):e0274893. doi: 10.1371/journal.pone.0274893 (PMC9521905; doi:10.1371/journal.pone.0274893)

**S2 Fig. Spatial variation in climate between the three genetic clusters.** Each point is a location along Principal Component scores (PC1 vs PC2) from a PCA on

the six bioclimatic variables overlapped in a bi-plot. PC1 and PC2 are primarily influenced by Temperature Seasonality (BIO4) and Precipitation of Driest Month

(BIO 14), respectively.


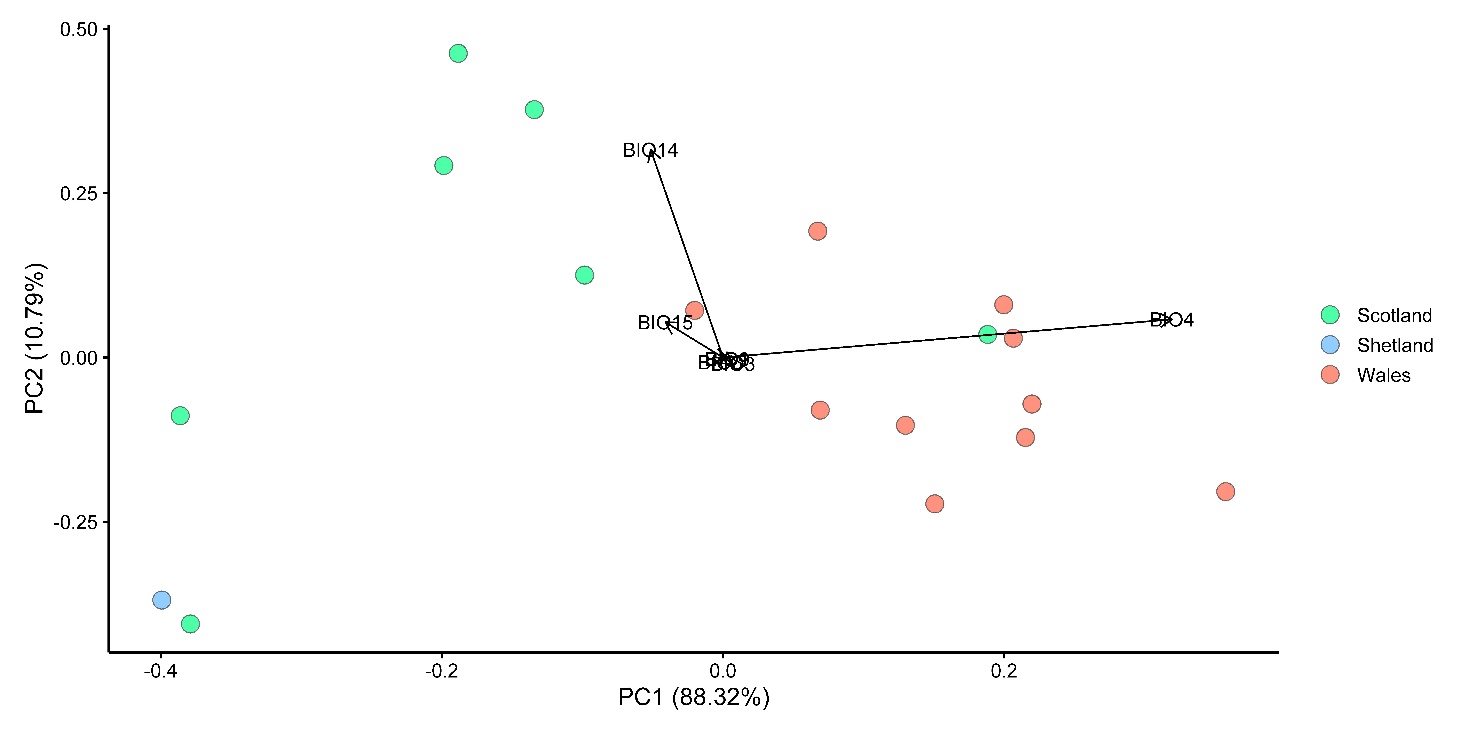

Supplement: S2 Fig — Each point is a location along Principal Component scores (PC1 vs PC2) from a PCA on the six bioclimatic variables overlapped in a bi-plot. PC1 and PC2 are primarily influenced by Temperature Seasonality (BIO4) and Precipitation of Driest Month (BIO 14), respectively. (DOCX) [file pone.0274893.s003.docx]
