# Supplementary material for: Better sturdy or slender? Eurasian otter skull plasticity in response to feeding ecology
Source: PLoS One. 2022 Sep 29;17(9):e0274893. doi: 10.1371/journal.pone.0274893 (PMC9521905; doi:10.1371/journal.pone.0274893)
Supplement: S1 Table — The sample is partitioned by sex for each geographic location. (DOCX) [file pone.0274893.s004.docx]

**S1 Table. Number of skull specimens analysed.** The sample is partitioned by sex for each geographic location.

| **Localities** | **Total sample** | **Female** | **Male** |
| --- | --- | --- | --- |
| Grampian | 6 | 3 | 3 |
| Mull | 3 | 0 | 3 |
| Orkney | 3 | 1 | 2 |
| Shetland | 15 | 8 | 7 |
| Skye | 1 | 1 | 0 |
| South Uist. | 1 | 0 | 1 |
| Strachclyde | 3 | 1 | 2 |
| Ullapool | 1 | 0 | 1 |
| Wales (Differents localities) | 10 | 3 | 7 |
