# Supplementary material for: Better sturdy or slender? Eurasian otter skull plasticity in response to feeding ecology
Source: PLoS One. 2022 Sep 29;17(9):e0274893. doi: 10.1371/journal.pone.0274893 (PMC9521905; doi:10.1371/journal.pone.0274893)
Supplement: S2 Table — Abbreviations: NMS = National Museums of Scotland, M = male, F = female, A = Adult. (DOCX) [file pone.0274893.s005.docx]

**S2 Table. Specimen list of the analysed otter skulls.** Abbreviations: NMS = National Museums of Scotland, M = male, F = female, A = Adult.

| **Museum** | **ID** | **Sex** | **Genetic clusters** | **Locality** | **Age** |
| --- | --- | --- | --- | --- | --- |
| NMS | Z-1995.004.051.pp | M | Scotland | Ullapool | A |
| NMS | Z-1995.004.053.pp | M | Scotland | Mull | A |
| NMS | Z-1998.085.030.pp | M | Scotland | Orkney | A |
| NMS | Z-1990.104.005.pp | M | Shetland | Shetland | A |
| NMS | Z-1998.085.022.pp | M | Scotland | Strachclyde | A |
| NMS | Z-1998.085.021.pp | M | Shetland | Shetland | A |
| NMS | Z-1998.085.027.pp | M | Shetland | Shetland | A |
| NMS | Z-1998.085.009.pp | M | Shetland | Shetland | A |
| NMS | Z-1998.085.016.pp | M | Shetland | Shetland | A |
| NMS | Z-1998.085.020.pp | M | Scotland | Mull | A |
| NMS | Z-1998.085.025.pp | F | Scotland | Skye | A |
| NMS | Z-1998.085.014.pp | F | Scotland | Grampian | A |
| NMS | Z-1998.085.024.pp | M | Scotland | Grampian | A |
| NMS | Z-1998.085.088.pp | F | Scotland | Orkney | A |
| NMS | Z-1998.085.031.pp | M | Scotland | Orkney | A |
| NMS | Z-1998.085.018.pp | F | Scotland | Strachclyde | A |
| NMS | Z-1995.004.058.pp | F | Shetland | Shetland | A |
| NMS | Z-1995.004.054.pp | F | Shetland | Shetland | A |
| NMS | Z-1990.104.021.pp | F | Shetland | Shetland | A |
| NMS | Z-1990.104.032.pp | F | Shetland | Shetland | A |
| NMS | Z-1990.104.013.pp | F | Shetland | Shetland | A |
| NMS | Z-1990.104.030.pp | F | Shetland | Shetland | A |
| NMS | Z-1990.104.010.pp | F | Shetland | Shetland | A |
| NMS | Z-1995.004.052.pp | F | Scotland | Grampian | A |
| NMS | Z-1998.085.003.pp | F | Scotland | Grampian | A |
| NMS | Z-1995.004.041.pp | M | Scotland | Grampian | A |
| NMS | Z-1998.085.042.pp | M | Scotland | Grampian | A |
| NMS | Z-1990.104.031.pp | M | Shetland | Shetland | A |
| NMS | CARDIFF-734 CU .pp | M | Wales | Wales | A |
| NMS | CARDIFF-696.pp | M | Wales | Wales | A |
| NMS | CARDIFF-793.pp | F | Wales | Wales | A |
| NMS | CARDIFF-676.pp | M | Wales | Wales | A |
| NMS | CARDIFF-723W.pp | F | Wales | Wales | A |
| NMS | CARDIFF-610.pp | M | Wales | Wales | A |
| NMS | CARDIFF-698.pp | M | Wales | Wales | A |
| NMS | CARDIFF-684.pp | M | Wales | Wales | A |
| NMS | CARDIFF-703.pp | M | Wales | Wales | A |
| NMS | CARDIFF-733.pp | F | Wales | Wales | A |
| NMS | Z-1998.106.pp | M | Scotland | Strachclyde | A |
| NMS | Z-1998.086.006.pp | M | Scotland | Mull | A |
| NMS | Z-1998.086.014.pp | M | Scotland | South Uist. | A |
| NMS | Z-1995.104.044.pp | F | Shetland | Shetland | A |
| NMS | Z-1990.104.029.pp | M | Shetland | Shetland | A |
