# Supplementary material for: Better sturdy or slender? Eurasian otter skull plasticity in response to feeding ecology
Source: PLoS One. 2022 Sep 29;17(9):e0274893. doi: 10.1371/journal.pone.0274893 (PMC9521905; doi:10.1371/journal.pone.0274893)
Supplement: S3 Table — Dietary data are based on. (DOCX) [file pone.0274893.s006.docx]

**S3 Table. Percentage of dietary items from 9 sample localities.** Dietary data are based on [1–8]**.**

**References**

1. Parry GS, Burton S, Cox B, Forman DW. Diet of coastal foraging Eurasian otters (Lutra lutra L.) in Pembrokeshire south-west Wales. Eur J Wildl Res. 2011;57: 485–494. doi:10.1007/s10344-010-0457-y

2. Yoxon P. Otter Surveys of the North West Scottish Islands. Sch Res Exch. 2008;2008: 1–10. doi:10.3814/2008/735403

3. Watt J. Seasonal and area‐related variations in the diet of otters Lutra lutra on Mull. J Zool. 1995;237: 179–194. doi:10.1111/j.1469-7998.1995.tb02757.x

4. Kruuk H, Moorhouse A. Seasonal and spatial differences in food selection by otters Lutra lutra in Shetland. J Zool. 1990;221: 621–637. doi:10.1111/j.1469-7998.1990.tb04021.x

5. Mason CF, Macdonald SM. The winter diet of otters (Lutra Zutra) on a Scottish sea loch. J Zool. 2009;192: 558–561. doi:10.1111/j.1469-7998.1980.tb04254.x

6. Carss N. Foraging behaviour and feeding ecology of the otter <em>Lutra lutra</em>: a selective review. Hystrix, Ital J Mammal. 1995;7: 179–194. doi:10.4404/hystrix-7.1-2-4069

7. Grant KR, Harrington LA. Fish selection by riverine Eurasian otters in lowland England. Mammal Res. 2015;60: 217–231. doi:10.1007/s13364-015-0223-3

8. McCluskie AE. Temperature-Mediated Shifts in the Foraging Behaviour of the Eurasian Otter, Lutra lutra L. Alan. 1999. Available: https://www.gla.ac.uk/myglasgow/research/enlighten/theses/digitisation/
