# Supplementary material for: Better sturdy or slender? Eurasian otter skull plasticity in response to feeding ecology
Source: PLoS One. 2022 Sep 29;17(9):e0274893. doi: 10.1371/journal.pone.0274893 (PMC9521905; doi:10.1371/journal.pone.0274893)
Supplement: S4 Table — Abbreviations: BIO = bioclimate. (DOCX) [file pone.0274893.s007.docx]

**S4** **Table. Definition of climatic variables used in the analyses.** Abbreviations: BIO = bioclimate.

| BIO1 = Annual Mean Temperature |
| --- |
| BIO2 = Mean Diurnal Range (Mean of monthly (max temp - min temp)) |
| BIO3 = Isothermality (BIO2/BIO7) (×100) |
| BIO4 = Temperature Seasonality (standard deviation ×100) |
| BIO5 = Max Temperature of Warmest Month |
| BIO6 = Min Temperature of Coldest Month |
| BIO7 = Temperature Annual Range (BIO5-BIO6) |
| BIO8 = Mean Temperature of Wettest Quarter |
| BIO9 = Mean Temperature of Driest Quarter |
| BIO10 = Mean Temperature of Warmest Quarter |
| BIO11 = Mean Temperature of Coldest Quarter |
| BIO12 = Annual Precipitation |
| BIO13 = Precipitation of Wettest Month |
| BIO14 = Precipitation of Driest Month |
| BIO15 = Precipitation Seasonality (Coefficient of Variation) |
| BIO16 = Precipitation of Wettest Quarter |
| BIO17 = Precipitation of Driest Quarter |
| BIO18 = Precipitation of Warmest Quarter |
| BIO19 = Precipitation of Coldest Quarter |
