# Supplementary material for: Better sturdy or slender? Eurasian otter skull plasticity in response to feeding ecology
Source: PLoS One. 2022 Sep 29;17(9):e0274893. doi: 10.1371/journal.pone.0274893 (PMC9521905; doi:10.1371/journal.pone.0274893)
Supplement: Attachment — Submitted filename: Response to Reviewers.docx [file pone.0274893.s008.docx]

We note that Figure 1 in your submission contain map/satellite images….

ANSWER: The map was obtained from [www.data.gov.uk](http://www.data.gov.uk) under the Open Government Licence v3.0 (<https://www.nationalarchives.gov.uk/doc/open-government-licence/version/3/>). We added this paragraph in the legend of Fig 1:

Country Boundaries was downloaded from www.data.gov.uk under the Open Government Licence v3.0

Academic Editor's (Bogdan Cristescu) Comments:
Two expert reviewers have provided positive assessment of the manuscript. Please address their comments in the revision.

ANSWER: Dear Editor, we are truly grateful for your suggestions that helped to greatly implement the manuscript and to clarify the contents of our study. Here following are detailed answers to the comments by the editor and the reviewers.

The Abstract is a bit long, please provide less detail especially with regard to methodology.

ANSWER: We modified the abstract according to your request.

Please consider a different color scheme for Fig. 3, as right now the colors coincide with some of the geographic localities from the other figures.

ANSWER: Done

For clarity of Figs. 5 and 7, please indicate what the skull schematics (edges and nodes) as well as view angles represent to justify their inclusion.

ANSWER: We added the following in the legends of fig 5 and 7:Below the x-axis are shown the wireframes corresponding to shape changes at the extremes of the axis. Nodes represent the landmarks, edges the anatomical connections between landmarks.

Reviewer #1: The authors did a great job with the methodology and results section. I had very few minor comments and two major comments for the discussion.

ANSWER: Dear Reviewer, thank you for your comments which have helped us improve our work

Minor Comments
Line 46: Add a comma between “mammals and skull shape” or change to “Several studies have shown that the skull shape in mammals is closely related…”

ANSWER: Done

Line 51: Add a comma between “expected” and “especially”.

ANSWER: Done

Line 62: Maybe removed the sentence “Here, we used 3D GM…” as this is stated again in the next paragraph when describing the objectives of the research. It sounded redundant.

ANSWER: Done

Line 96: What is the fixed distance on the tripod?

ANSWER: We modified the sentence: placed on the tripod at a fixed distance (50 cm) from the turntable

Line 103: Add a space between “data” and “[41,47]”.

ANSWER: Done

Line 114: Add a period after “[48,49]”

ANSWER: Done

Line 125: Add a comma between “size or shape” and “we used…”

ANSWER: Done

Line 140: Add space between “factors” and “[56]”

ANSWER: Done

Line 141: Be consistent with subtitles. I recommend using “Shape and climate.” to be consistent with the “Shape and diet.” in line 128.

ANSWER: Done

Line 156: Add “being” between “always” and “larger”… “with male skulls always being larger than female’s…”

ANSWER: Done

Line 224: Change “an” to “and” and put a comma after “Shetland”?

ANSWER: Done

Line 225: Remove “,” after (BIO4). Capitalize “the”. I believe this is a new sentence.

ANSWER: Done

Line 225: Remove the space between “values” and the period.

ANSWER: Done

Line 226: “scottiesh”?

ANSWER: We replace “scottiesh” with “Scottish”

Line 248: Add a space between “sizes” and “[25,67]”

ANSWER: Done

Line 253: Add space between “morphometry” and “[69]”

ANSWER: Done

Line 269: Change “durophage” to “durophagous”.

ANSWER: Done

Line 291: Name the authors for reference 14, like Stanton et al. 2014 to be consistent.

ANSWER: Done

Line 294: Remove space between “resources” and the period.

ANSWER: Done

Major Comments: I would like to see more information in the discussion section.
Line 51-53: The authors mention that cranial and mandibular morphology are expected to have modifications in dentition and masticatory muscle attachment area. It would be a nice addition to include hypotheses in how these morphological modifications affect the masticatory muscles in the discussion based on diet. A lot can be discussed on muscle attachment and the feeding biomechanics per group based on dietary differences.

ANSWER: Thanks for your suggestion, we added this paragraph at line 286: Nevertheless, a more elongated braincase allows attachment for posterior and anterior temporalis muscles, increasing the horizontal force [77] ; and this paragraph at line 289 : The wider zygomatic arches and taller crania in marine feeders can allow an increased area for the attachment of the masseter and temporalis jaw adductor muscles [30,78]. These muscles function primarily to close the jaw [77] and the resulting larger temporalis mass in marine feeders can allow a stronger bite force [78,79]

The authors showed that there was variation among skull morphology based on climate, but it is not well discussed in the discussion area. It would be a great addition to the overall manuscript if this was better explained in the discussion with more detail. I was interested in knowing more details about climate variation among the groups and thought it would be better explained in the discussion. How did climate specifically affect the morphology between the groups?

ANSWER: We added the following paragraph to the discussion in line 311. Skull traits associated with diet variation were similar to the traits associated with climate variation, as confirmed by comparison of PLS results. Climate is commonly used as a proxy of diet when this information is not available (see [52,62,89]) and our evidence suggests that climate may be used as a proxy for diet adaptation in the Eurasian otter also. On the other hand, Tseng and Flynn [90] indicated that skull shape variation in carnivores is correlated with precipitation, as this latter drives modifications in the sensory systems. In our case, the larger nasal cavity observed in otters living in the coldest areas, could increase oxygen assumption. This association is supported by evidence from Yom-Tov et al. [91] which shows negative association between water temperature and oxygen consumption in Eurasian otters

Reviewer #2: Manuscript review
Better sturdy or slender? Eurasian otter skull plasticity in response to feeding ecology and climate
Line 49: Add cite to the sentence.

ANSWER: Done

Line 96: Change the citation (Loy et al. 2021) to the journal numeric format.

ANSWER: Done

Line 131: Change (Suppl. Mat. 4) for (Suppl. Mat. 3).

ANSWER: Done

Line 141. Change (Suppl. Mat. 3) for (Suppl. Mat. 4).

ANSWER: Done

Line 189. Change (Suppl. Mat. 4) for (Suppl. Mat. 5).

ANSWER: Done

Line 223. Change (Suppl. Mat. 5) for (Suppl. Mat. 6).

ANSWER: Done

Introduction:
This sentence is a bit repetitive with the paragraph in line 71: “Here, we used 3D GM of the skull to investigate the morphological variation of otters across the mainland and the islands of Great Britain, and to explore the ultimate drivers of the observed patterns in terms of the genetic, latitudinal and ecological differentiation revealed by recent studies”. Change the wording so that the information does not sound repetitive.

ANSWER: Done
Supplementary material:
The legend of Supp. Mat. 6 need check the redaction and format.

Questions
All your specimens were adult individuals or did you not consider that variable? since it can affect the results, especially in the variation in size between males and females.

ANSWER: Yes they are all adults, as reported at line 89 in the methods

It is true that they did not find a relationship with Bergman's rule, but they did not consider that the specimens that lives in Shetland are on an island far from the mainland and that several species of mammals that inhabit islands have dwarfism or smaller sizes than their congeners. who live on mainland.

ANSWER: Thanks for your comment, we added this paragraph in the line 276 : [72,75]. The Shetland specimens could be subject to the island rule (i.e. smallest size [38]) and bias the gradient in size predicted by Bergmann rules, in fact, the lnCS of Scotland population seems to be larger than Wales.

I suggest that you further enrich the discussion of paragraph 273, I think you could cite works related to climatic-diet-morphology variation to further support your discussion.

ANSWER: We added the following paragraph to the discussion in line 311. Skull traits associated with diet variation were similar to the traits associated with climate variation, as confirmed by comparison of PLS results. Climate is commonly used as a proxy of diet when this information is not available (see [52,62,89]) and our evidence suggests that climate may be used as a proxy for diet adaptation in the Eurasian otter also. On the other hand, Tseng and Flynn [90] indicated that skull shape variation in carnivores is correlated with precipitation, as this latter drives modifications in the sensory systems. In our case, the larger nasal cavity observed in otters living in the coldest areas, could increase oxygen assumption. This association is supported by evidence from Yom-Tov et al. [91] which shows negative association between water temperature and oxygen consumption in Eurasian otters
